# Safety and efficacy of Holmium-166 selective internal radiotherapy of primary and secondary liver cancer confirmed by real-world data

**DOI:** 10.3389/fonc.2024.1404621

**Published:** 2024-06-11

**Authors:** Victor Schulze-Zachau, Gontran Verset, Pieter De Bondt, Katrien De Keukeleire, Falk Gühne, Martin Heuschkel, Ralf-Thorsten Hoffmann, Elena Bozzi, Rosa Sciuto, Marnix Lam, Jordi Deportós Moreno, Roxane Debrus, Christoph J. Zech

**Affiliations:** ^1^ Radiology and Nuclear Medicine Clinic, University Hospital Basel, Basel, Switzerland; ^2^ Hôpital Universitaire de Bruxelles (HUB)-Hôpital Erasme, Université Libre de Bruxelles, Brussels, Belgium; ^3^ Department of Nuclear Medicine, Onze-Lieve-Vrouwziekenhuis (OLV) Aalst, Aalst, Belgium; ^4^ Radiology Department, Algemeen Stedelijk Ziekenhuis (ASZ) Aalst, Aalst, Belgium; ^5^ Jena University Hospital, Clinic of Nuclear Medicine, Jena, Germany; ^6^ Nuclear Medicine Clinic, Rostock University Medical Center, Rostock, Germany; ^7^ Diagnostic and Interventional Radiology Institute, University Hospital Carl Gustav Carus Technische Universität (TU) Dresden​, Dresden, Germany; ^8^ Interventional Radiology Department, University Hospital Pisa, Pisa, Italy; ^9^ Nuclear Medicine Clinic, Istituti Fisioterapici Ospitalieri (IFO) Regina Elena Hospital Roma​, Rome, Italy; ^10^ Nuclear Medicine Clinic, University Medical Center Utrecht, Utrecht, Netherlands; ^11^ Nuclear Medicine Clinic, Hospital Germans Trias i Pujol, Barcelona, Spain; ^12^ Terumo Europe, Leuven, Belgium

**Keywords:** selective internal radiotherapy, transarterial radioembolization, Holmium-166, Holmium-166 microspheres, hepatic malignancy

## Abstract

**Purpose:**

Holmium-166 has emerged as a promising option for selective internal radiotherapy (SIRT) for hepatic malignancies, but data on routine clinical use are lacking. The purpose of this study was to describe the safety and effectiveness of Holmium-166 SIRT in real-world practice through retrospective analysis of a multicenter registry.

**Methods:**

Retrospective analysis was conducted on Holmium-166 SIRT procedures performed between July 15, 2019, and July 15, 2021, across seven European centers. Treatment planning, treatment realization and post-treatment follow-up were conducted according to routine local practice. Safety and effectiveness data were extracted from the patients’ health records. Primary endpoint analysis was assessed for the entire study population with separate analysis for subgroups with hepatocellular carcinoma, metastatic colorectal cancer and intrahepatic cholangiocarcinoma.

**Results:**

A total of 167 SIRT procedures in 146 patients (mean age 66 ± 11 years, 68% male) were retrospectively evaluated. Most common tumor entities were hepatocellular carcinoma (n=55), metastatic colorectal cancer (n=35), intrahepatic cholangiocarcinoma (n=19) and metastatic neuroendocrine tumors (n=10). Nine adverse events grade ≥ 3 according to Common Terminology Criteria for Adverse Events were recorded, including one fatal case of radioembolization-induced liver disease. Response rates and median overall survival for the above mentioned subgroups were comparable to results from previous Holmium-166 trials as well as to results from Yttrium-90 registries.

**Conclusion:**

This study confirms that the safety and effectiveness of Holmium-166 SIRT derived from prospective trials also applies in routine clinical practice, reinforcing its potential as a viable treatment option for primary and secondary liver cancer.

## 1 Introduction

Selective internal radiotherapy (SIRT), also known as trans-arterial radioembolization (TARE), is a minimally invasive procedure during which radioactively-loaded microspheres are injected into the hepatic arteries in order to treat hepatic malignancies. According to guidelines, SIRT represents a treatment modality for patients with only or predominantly hepatic disease not suitable for surgery or ablative therapy or with failure of systemic therapy or inacceptable side effects (1–5). Hepatocellular carcinoma (HCC), intrahepatic cholangiocarcinoma (iCC) and hepatic metastases, e.g. from colorectal cancer (mCRC), are typical target entities. Before performing therapeutic SIRT, treatment planning is carried out with a reduced activity in order to simulate distribution of the therapeutic dose and to detect potential pulmonary or gastrointestinal shunting. Recent literature underlines the potential suitability of SIRT for individualized medicine (6, 7) and the development of radiation segmentectomy (8).

While Yttrium-90 (^90^Y) represents the conventional isotope for SIRT with technetium-99m macroaggregated albumin (^99m^Tc-MAA) used for planning the treatment, an alternative platform based on Holmium-166 (^166^Ho) has been developed since the 1990s. ^166^Ho microspheres for SIRT are commercially available as QuiremSpheres™ (Quirem BV, Deventer, the Netherlands), and QuiremScout™ (Quirem BV, Deventer, the Netherlands) (9). The use of ^166^Ho offers interesting advantages: treatment planning and treatment can be performed using identical microspheres, which reduces discrepancy between both procedures and allows for optimized predictability of the distribution of the therapeutic injection (10) and improved prediction of pulmonary uptake of the activity (11). Furthermore, the element Holmium is a chemical lanthanide with paramagnetic properties, which enables quantification of hepatic ^166^Ho dose via MRI (9). This offers the possibility of intraprocedural MRI-based dosimetry, which has recently shown to be feasible (12). Following the administration of the scout dose, SPECT imaging of the thorax and abdomen is conducted in order to detect unintended delivery to the lungs or upper abdominal organs. The SPECT images obtained hereby serve as comparison to the post-treatment images in order to assess activity deposition outside the liver, the percentage of activity that has reached the lungs, the uniformity of the dose distribution, and the ratio of absorbed dose between the tumor and healthy tissues. For ^166^Ho SIRT, dedicated software (Q-suiteTM, Quirem BV, Deventer, The Netherlands) is available both for treatment planning and for dose reconstruction during treatment evaluation.

Several studies agreed on general safety and efficacy of ^166^Ho SIRT for various hepatic malignancies (13–16). However, recent publications point out the value of so called real-world evidence in addition to the findings derived from controlled trials (17). Real-world data can be defined as comprising any data not acquired with a primary scientific intention, such as electronic health records. Information on patient subgroups underrepresented in prospective trials may be available in real-world data and rare or late occurring side effects may be revealed. Furthermore, real-world data can help to analyze and evaluate the process of implementation of healthcare innovations into clinical routine.

The objective of this real-world, multi-center, retrospective registry was to describe the safety and effectiveness of ^166^Ho SIRT in real-world practice.

## 2 Materials and methods

### 2.1 Inclusion criteria

All consecutive patients treated with 166-Ho-SIRT for primary liver tumors or metastasis between July 15, 2019 and 15 July, 2021 in seven participating European centers were included. The study has been approved by the responsible ethical boards and has received the institutional approval number BASEC-Nr 2021–02357. Treatment allocation decisions were made by a local interdisciplinary tumor board. In Europe, SIRT is typically indicated for the treatment of primary and metastatic liver tumors not amenable to resection or ablation. ^166^Ho and ^90^Y SIRT have similar indications and contraindications. Contraindications include life expectancy of less than three months, pregnancy, clinical liver failure, disseminated extrahepatic disease and extrahepatic spread of radiation dose predicted by the treatment planning procedure.

Patients previously included in prospective studies with ^166^Ho SIRT were not included. Data collection took place between December 2021 and March 2022.

### 2.2 Treatment

Pre-treatment work-up and the treatment itself were performed according to routine practice in the study sites. Both ^99m^Tc-MAA or ^166^Ho Scout could serve as surrogate marker during the treatment planning procedure. Treatment was performed in a single session or in multiple sessions. A recommendation that the absorbed whole liver radiation dose should not exceed 60 Gy has been developed. Guidelines for conducting the SIRT procedure were developed by European Association of Nuclear Medicine (EANM) (3).

The technical performance of SIRT was evaluated through pre- and post-treatment dosimetry and clinical practice descriptors: administered activity, liver volume, treatment approach (selective, lobar, whole liver), treatment volume, dosimetry outcomes in terms of target and normal liver dose (either recorded from post-treatment imaging or based on injected activity and target volume). Software used to perform the treatment planning and treatment procedures could be conventional software used at the study site or Q-Suite™. Calculation of treatment activity and post-treatment dose evaluations were performed according to local clinical routine.

The typical imaging techniques employed for dose estimation (work-up SPECT imaging of surrogates ^99m^Tc-MAA or ^166^Ho Scout and MR imaging of therapeutic ^166^Ho microspheres) have been discussed in previous studies (8, 17). The process of voxel-level dosimetry using Q-Suite is outlined in the manufacturer’s instructions for use (https://www.quirem.com/ifu/). In short, SPECT-based dosimetry involves two main steps of calibrating the activity map and reconstructing the dose map. During dose estimation of work-up, first, the SPECT image is scaled to the intended activity for each target liver. Then, using conversion factor of 16 [MBq/J], absorbed dose is derived from activity map. For assessing the dose after treatment using SPECT image, first either a pre-established system calibration or a patient-specific calibration factor (based on the total counts in a user-defined volume and the total administered activity) is used to convert counts to Bq. After calibration, an absorbed dose map is reconstructed, either based on a local deposition model or a pre-defined dose-point kernel. MR-based dose evaluation after treatment is described in detail elsewhere (18). The imaging techniques employed in this retrospective observational study were dependent on the investigator’s choice and routine clinical practice.

### 2.3 Data collection

At each participating institution, a retrospective search of the patients’ electronic health records was conducted including the clinical information system (CIS), the picture archiving and communication system (PACS) and the laboratory information system (LIS), which usually contained data from pathologic examinations, too. The search followed a predefined list of variables. If certain variables could not be obtained in the electronic health records of the institution, the search was expanded to external health care providers such as general practitioners.

The following data were extracted: patient baseline characteristics, pre-treatment patient status, SIRT work-up procedure(s), SIRT procedures, post-treatment evaluation, occurrence of adverse events of specific interest (AESIs) and of adverse events (AEs) grade 3 or higher according to CTCAE (Common Terminology Criteria for Adverse Events) version 5.0. Th.

### 2.4 Safety assessment

Safety primary endpoint analysis was performed for short term (30 days), median term (1–12 months), long term (>12 months), and overall safety (at any time). The primary safety endpoint was defined in terms of reported incidence of AEs according to CTCAE grade ≥ 3, or any of the following AESIs known to be associated with SIRT regardless of their CTCAE grading: acute pancreatitis, gastric ulceration, gastritis, radiation pneumonitis, radioembolization-induced liver disease (REILD), and cholecystitis. Safety assessment was also performed based on routine blood tests with an emphasis on the liver function parameters obtained at baseline, after the SIRT procedure and throughout the follow-up period of 12 months with specific time windows of 1 week, 3, 6, 9, and 12 months.

### 2.5 Efficacy assessment

Progression Free Survival (PFS) was defined as the time from first SIRT procedure until overall progression or death. Hepatic Progression Free Survival (hPFS) was defined as the time from first SIRT procedure until hepatic progression or death. Overall survival (OS) was defined as the time from first SIRT procedure until death from any cause.

Tumor response in the liver according to mRECIST and/or RECIST 1.1 was analyzed at 3 months (+/- 14 days) after SIRT had been completed, or beyond the 3-months point, depending on the available information, and evaluated as best overall response. Disease control (DC) rate was defined as the sum of objective response (complete or partial response) and stable disease rates.

### 2.6 Statistical analyses

Patient demographics, baseline characteristics, and procedure characteristics were summarized with mean, standard deviation, median and range for continuous variables and with frequencies, percentages, and 95% confidence intervals for discrete variables.

Treatment response was reported as percentage of all patients with known outcome. For time to event clinical endpoints, Kaplan-Meier survival curves were presented.

All analyses were conducted using SAS v9.4 (SAS Institute, Cary, NC) or the latest available SAS software.

### 2.7 Ethical considerations

The study was approved by the Ethics Committees of all participating institutions. Due to the retrospective nature of the study, a waiver of informed consent was requested to the Ethics Committees in charge and approved.

## 3 Results

### 3.1 Patient characteristics and data collection

**Figure 1:**
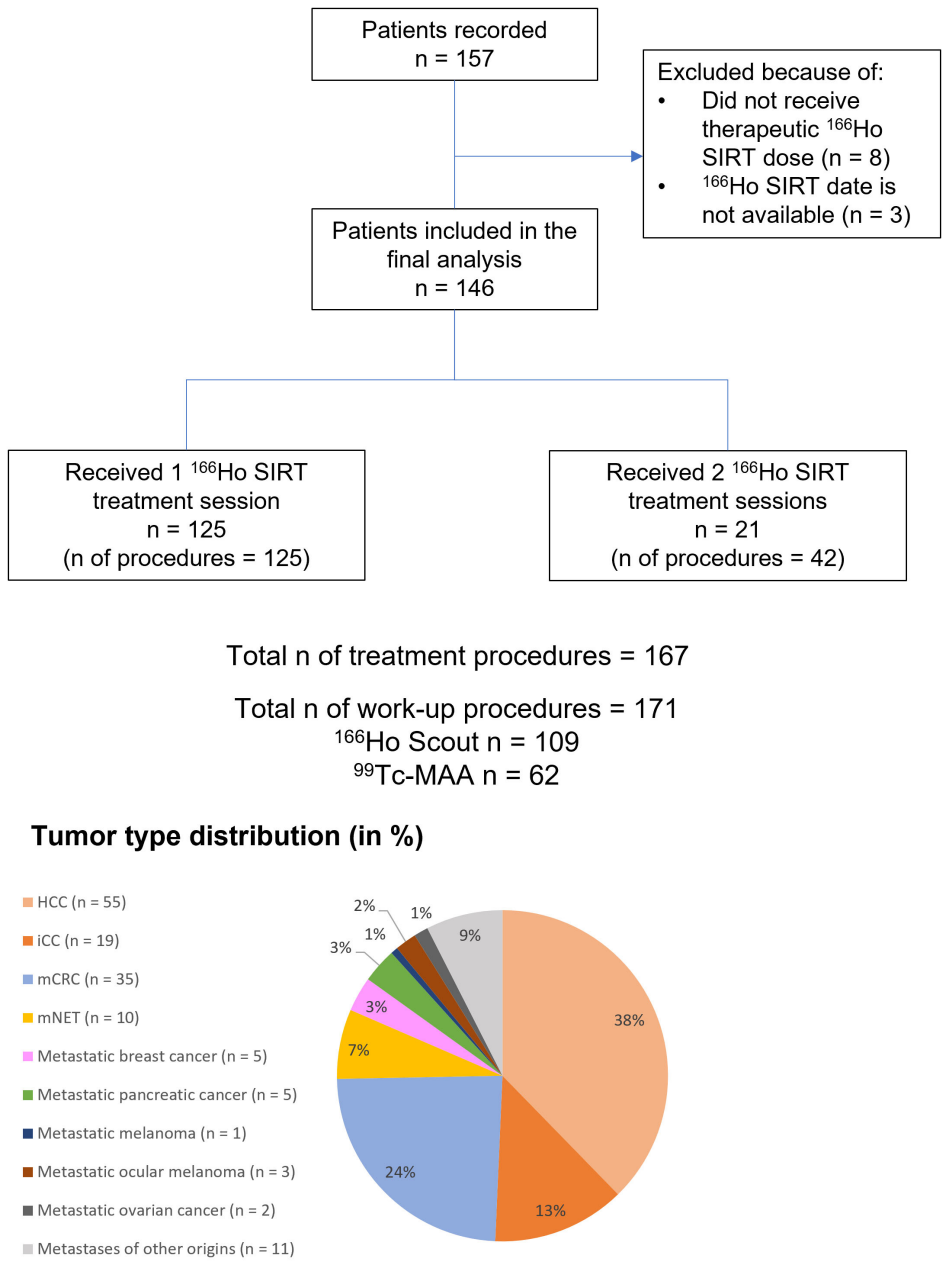
Flow chart of the study. HCC, hepatocellular carcinoma; iCC, intrahepatic cholangiocarcinoma; mCRC, metastatic colorectal cancer; mNET, metastatic neuroendocrine tumor; SIRT, selective internal radiotherapy.

A total of 157 patients were recorded, including eight patients who did not receive the therapeutic dose due to unfavorable outcomes of the treatment planning procedure, and three patients who received the therapeutic dose but for whom treatment date was not available, which precluded time to event analysis. 146 patients from seven participating centers were included in the final analysis (**Figure 1**). These included 55 cases of HCC, 19 iCC, 35 mCRC, and 37 liver metastases of other origins. Baseline characteristics of the study population and treatment intent are presented in **Table 1**. Disease characteristics of the 55 patients with HCC are presented in **Table 1**. Among the 19 patients with iCC, 9 patient (47%) were treated with palliative intent, 1 (5%) with curative intent, 3 (16%) were treated for downstaging and another 3 (16%) – for bridging to resection.

**Table 1:** Patient baseline and treatment characteristics.

Baseline characteristics, full cohort, N/N with available data (%)	
Sex (male)	99/146 (67.8%)
Age (years, mean)	66.2 ± 10.9
Eastern Cooperative Oncology Group (ECOG) score	
ECOG 0	59/146 (40.9%)
ECOG 1	46/146 (31.5%)
ECOG 2	8/146 (5.5%)
Unknown	33/146 (22.6%)
Total bilirubin, mean (SD)	95/146 (65.1%), 14.01 (16.95) µmol/L
Tumor distribution	
Bilobar	92/146 (63.0%)
Unilobar, > 1 segment	26/146 (17.8%)
Segmental	21/146 (14.4%)
Unknown	7/146 (4.8%)
Extrahepatic disease	39/146 (26.7%)
Baseline characteristics patients with HCC, N/N with available data (%)	
Tumor distribution	
Bilobar	35/55 (63.6%)
Unilobar, > 1 segment	13/55 (23.6%)
Segmental	7/55 (12.7%)
Extrahepatic disease	9/55 (16.4%)
Portal vein invasion	6/55 (10.9%)
BCLC stage	
BCLC 0	2/55 (3.6%)
BCLC A	3/55 (5.5%)
BCLC B	32/55 (58.2%)
BCLC C	11/55 (20.0%)
Unknown	7/55 (12.7%)
Child Pugh status	
Child Pugh status A	36/55 (65.5%)
Child Pugh status B	17/55 (30.9%)
Child Pugh status C	2/55 (3.6%)
Total bilirubin, mean (SD)	36/55 (65.5%), 14.20 (8.7) µmol/L
Treatment characteristics, full cohort, N/N with available data (%)	
Treatment intent	
Palliative	97/146 (66.4%)
Alternative option to curative treatment	24/146 (16.4%)
Bridge to transplant	11/146 (7.5%)
Downstage to resection	5/146 (3.4%)
Bridge to resection	3/146 (2.1%)
Other/missing	6/146 (4.1%)
Number of treatments	
1	125/146 (85.6%)
2	21/146 (14.4%)
Mean N of treatments	1.1 ± 0.35
Treatment planning characteristics*	
^166^Ho Scout used	109/171 (63.7%)
^99m^Tc-MAA used	62/171 (36.3%)
Total administered activity, mean ± SD (GBq)	4.1 ± 2.25
Whole liver dose (Gy)	37.6 ± 16.58 (101)
Above target (60 Gy) liver absorbed dose	6/167 (3.6%)
Predicted tumor absorbed dose (Gy)	155.6 ± 97.40 (59)
Predicted non-tumor absorbed dose (Gy)	36.4 ± 15.85 (52)
Procedure characteristics	
Selective	38/167 (22.8%)
Lobar	84/167 (50.3%)
Bilobar	24/167 (14.4%)
Whole liver	16/167 (9.7%)
Unknown	5/167 (3.0%)
Tumor absorbed dose (Gy)	117.0 ± 87.26 (n=56)
Non tumor absorbed dose (Gy)	31.9 ± 15.56 (n=53)

*Predicted target and non-target (normal liver absorbed) dose are available for all patients, not just those for whom treatment planning was performed using Ho-166 Scout.

### 3.2 Treatment

A total of 167 treatment procedures were performed. Of all patients, 85.6% (125) received a single treatment session with ^166^Ho SIRT, and 14.4% (21) received two treatment sessions with ^166^Ho SIRT (**Figure 1**). The median number of days between the first and second treatment session was 43 days (range 8–354 days). No patients received 3 or more treatments. Details on treatment planning are provided in **Table 1** and **Figure 1**.

Average whole liver absorbed dose was available for 101 patients, of whom 95 had a whole liver average absorbed dose < 60 Gy. Six patients (5.9%) exceeded the 60 Gy whole liver recommendation: three patients by < 10 Gy, and three patients had a whole liver absorbed dose of between 80 and 90 Gy. For further details of post-treatment SPECT, see **Table 1**.

### 3.3 Tumor response

**Figure 2:**
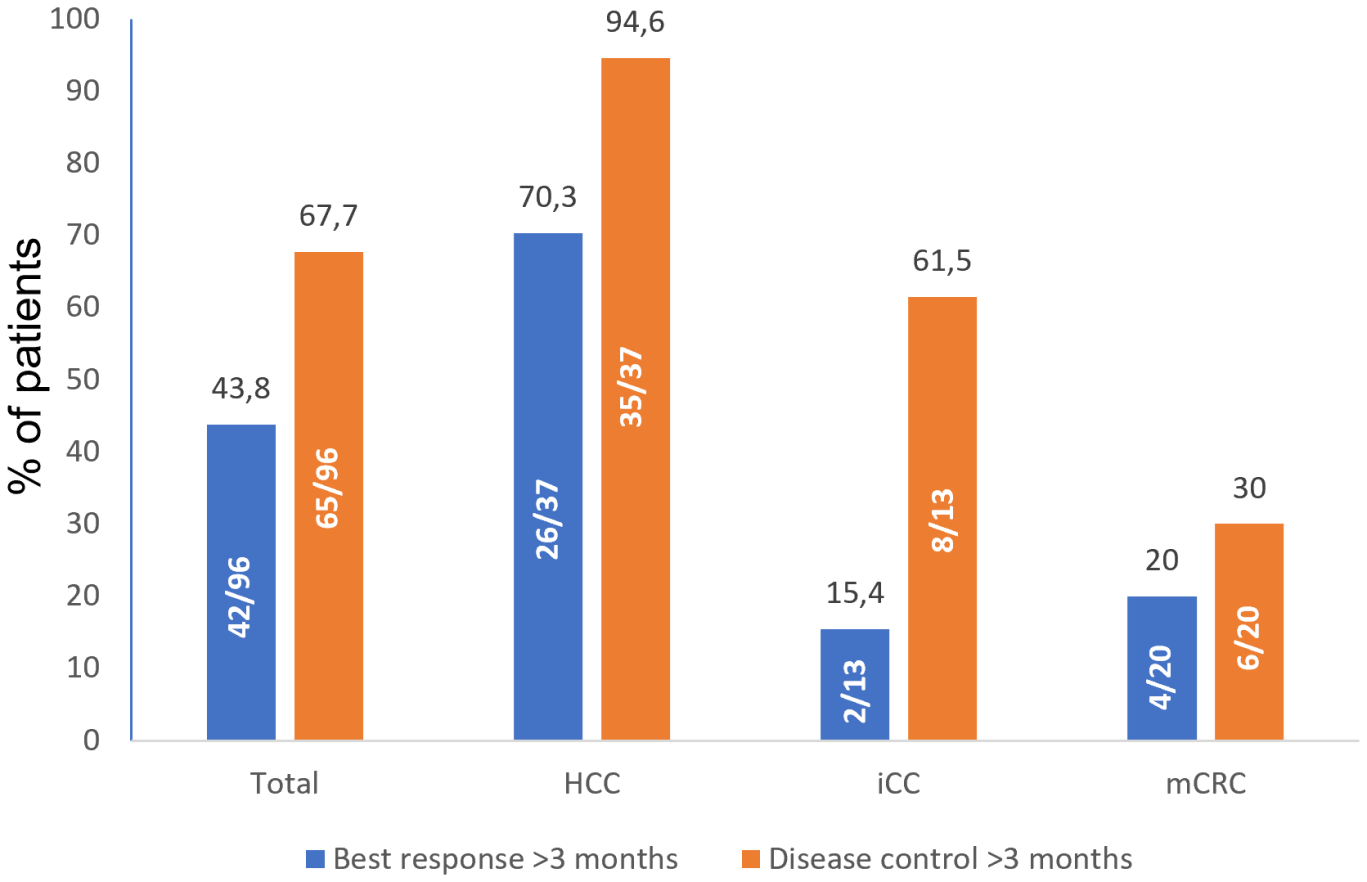
Best response and disease control rates (%) beyond three months. Best response was defined as combination of tumor responses in target and non-target lesions; disease control was defined as combination of complete response, partial response, and stable disease. HCC, hepatocellular carcinoma; iCC, intrahepatic cholangiocarcinoma; mCRC, metastatic colorectal cancer.

**Figure 3:**
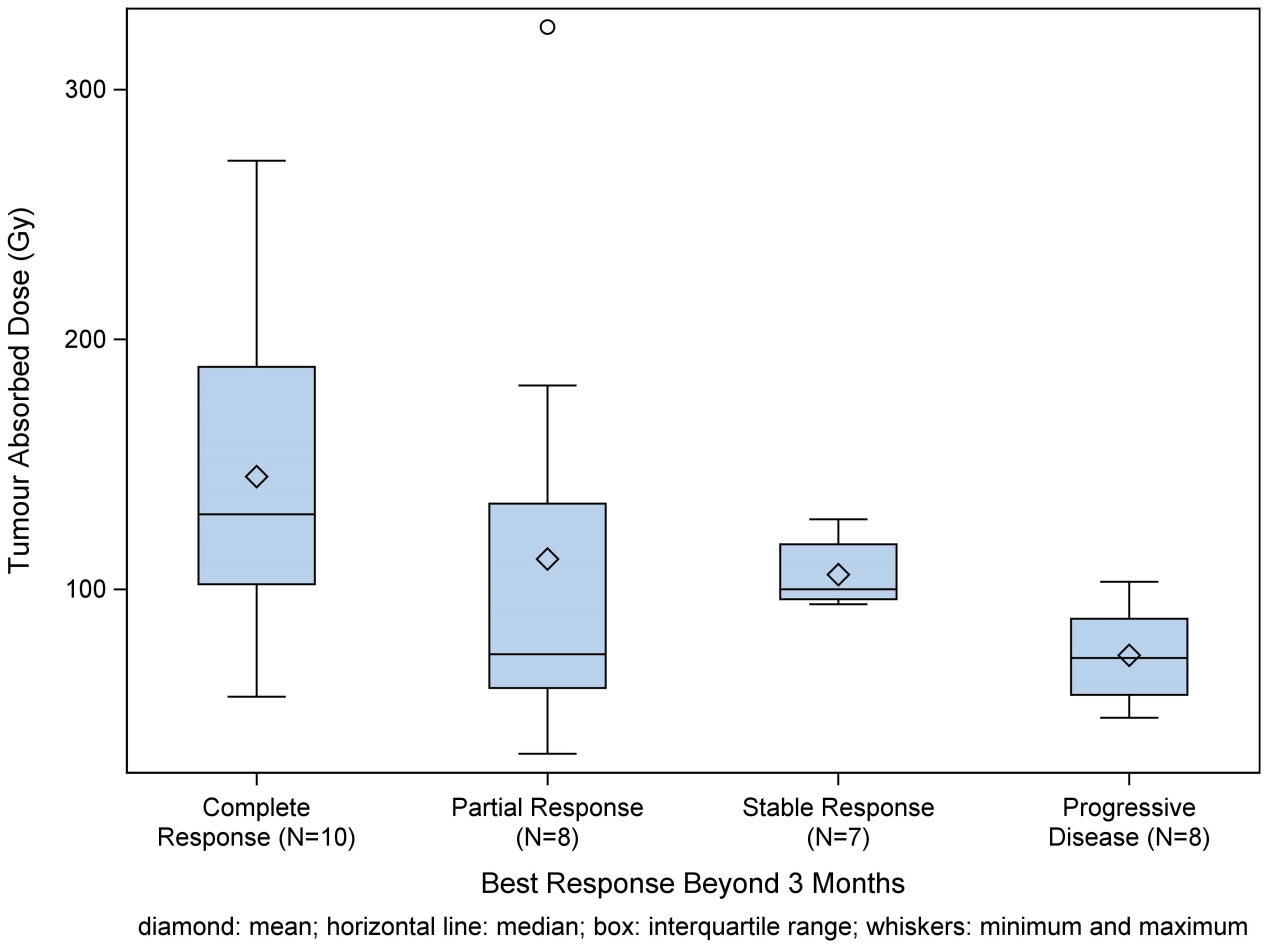
Box plot of tumor absorbed dose per best response category beyond three months shows a non-significant trend towards dose-response-relationship. Best response was defined as combination of tumor responses in target and non-target lesions; disease control was defined as combination of complete response, partial response, and stable disease.

Among the patients with available tumor response assessment criteria, mRECIST was used in 48 cases, and RECIST 1.1 was used in 30 cases. For evaluation at three months after SIRT (+/- 14 days), response assessment was available for 39.7% (58 evaluated) of patients (**Table 2**). Best response and disease control rates beyond three months in different subgroups per diagnosis are presented in **Figure 2**. Tumor absorbed dose per best response beyond three months is presented on **Figure 3**.

**Table 2:** Tumor response rate and treatment outcomes, all subgroups.

Clinical Endpoints	n/N with data available (%)
Response rate at 3 months	21/58 (36.2)
Disease control rate at 3 months	37/58 (63.8)
Disease progression rate at any time	71/146 (48.6)
Hepatic progression rate at any time	63/146 (43.2)
Liver transplantation rate after SIRT	4/146 (2.7)
Liver resection rate after SIRT	6/146 (4.1)

### 3.4 Survival

Survival outcomes were analyzed for patients with HCC (n=55), mCRC (n=35) and iCC (n=19). Patients were followed for a median of 7.1 months (range 0.1- 26.4 months]. The proportion of censored patients was 54.1% for OS, 33.8% for PFS and 37.9% for hPFS.

Median PFS was 5.3 months (95% CI 3.8–7) in the total evaluable population, of which 33.8% (49) of patients were censored. Median PFS was 9.1 months in HCC (95% CI 7.1–14), 3.2 months in mCRC (95% CI 2.8-n.e. (non-estimable)), 3.9 months in iCC (95% CI 3.0–8).

**Figure 4:**
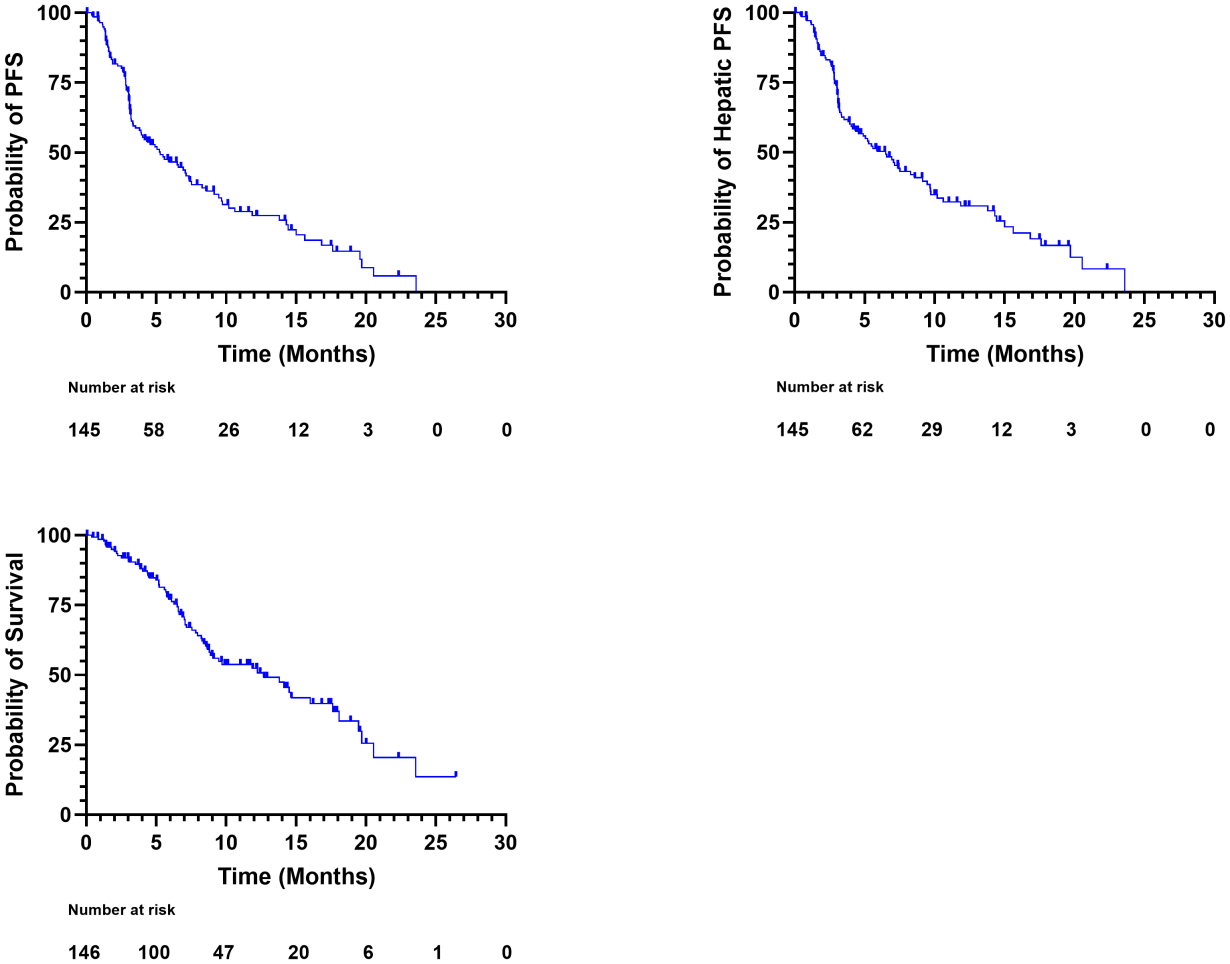
Kaplan-Meier plots of progression free survival (upper left), hepatic progression free survival (hPFS) (upper right) and overall survival (OS) (lower left) for the total population.

Overall median hPFS was 6.5 months (95% CI 4.1, 9) (**Figure 4**), with 37.9% (55) of patients in this population censored. Median hPFS was 9.7 months in HCC (95% CI 7.1–14), 3.2 months in mCRC (95% CI 2.8–5) and 6.6 months in iCC (95% CI 3.0–8).

Overall median OS was 12.7 months (95% CI 8.8–18) (**Figure 4**). 54.1% (79) of the total population was censored. Median OS was 14.7 months in HCC (95% CI 13.8-n.e.), 8.9 months in mCRC (95% CI 7.1–14) and 8.3 months in iCC (95% CI 6.6–24), respectively.

### 3.5 Hepatocellular injury markers

**Figure 5:**
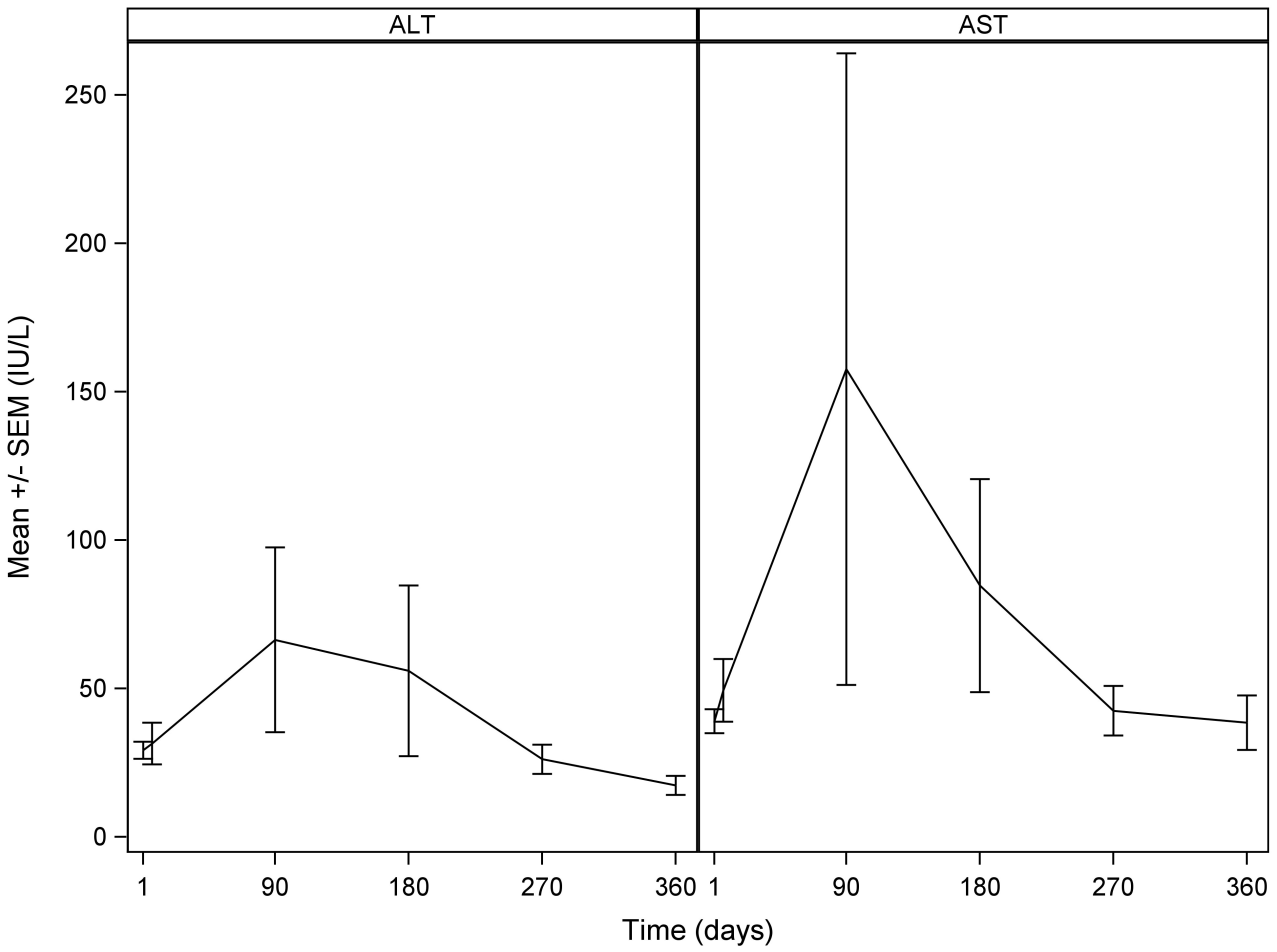
Mean alanine aminotransferase (ALT) (left) and aspartate transaminase (AST) (right): mean ± standard deviation with standard error over time. Time in days refers to the number of days after the SIRT treatment.

Both AST and ALT demonstrated a post-treatment increase. Mean AST increased from 38.99 ± 37.42 IU/L (mean ± SD) at baseline to the maximal value of 157.63 ± 796.61 IU/L at 90 days. Mean ALT increased from 29.29 ± 27.38 IU/L at baseline to 66.42 ± 237.22 IU/L at 90 days (**Figure 5**). Both parameters returned to baseline levels (42.52 ± 48.74 IU/L for AST and 26.17 ± 29.65 IU/L for ALT) at 270 days (9 months). Bilirubin levels showed a gradual increase from the baseline value of (mean ± SD) 1.763 ± 12.82 µmol/L to the maximum value of 260.611 ± 994.45 µmol/L at 180 days, returning back to 181.546 ± 641.91 µmol/L at 270 days and 51.303 ± 75.91 µmol/L at 360 days (12 months).

### 3.6 Safety

Among the three patients who received a 80–90 Gy liver absorbed dose, one reported epigastric pain as an AE with onset 31 days after the procedure with possible relation to the procedure, which later resolved. In five (3.4%) patients, at least one AESI was reported. Acute pancreatitis, or radiation pneumonitis were not reported. Gastric ulceration was reported in 2.1% (3) of patients (**Table 3**). A total of five fatal AEs were recorded in the study, three of them possibly or probably related to the treatment device and/or procedure. One fatal case of cholecystitis with onset of 32 days after the procedure was reported, and assessed as possibly related to the procedure and the treatment device. REILD was reported in one patient (0.7%) with an onset of 89 days after the first, and 39 days after the second treatment, which resulted in the patient’s death. A probable relation to the procedure and possible relation to the device was reported. In this case, SIRT was performed with a palliative intent in a patient with bilobar HCC, BCLC stage B, Child Pugh score A with slightly elevated total bilirubin of 30 µmol/l at baseline. No extrahepatic disease or portal vein invasion was reported. Separate treatments of left and right liver lobe were performed at an interval of two months with average absorbed lobar doses of approximately 55 Gy and 60 Gy, respectively. One case of acute or chronic renal failure was recorded 91 days after the treatment, and resulted in the patient’s death. This case was assessed as possibly related to the treatment procedure, but not to the device.

**Table 3:** Adverse Events reported by toxicity grade and day of onset.

Reported AE	Day of Onset*	Relation to Procedure^$^	Relation to Device^$^	ADE	SADE	USADE	Serious	Toxicity Grade^#^
REILD	89	Probable	possible	Y	Y	N	Y	5
acute or chronic renal failure	91	possible	not related	N	N	N	Y	5
tricuspid valve disease	558	not related	not related	N	N	N	Y	4
post-embolization syndrome	1	causal	causal	Y	N	N	N	3
abdominal pain	20	possible	possible	Y	Y	N	Y	3
epigastric pain	32	possible	possible	Y	N	N	N	3
cholecystitis	32	possible	possible	Y	Y	N	Y	3
dyspnea	176	not related	not related	N	N	N	Y	3
myocardial infarct	490	not related	not related	N	N	N	Y	3
Gastric Ulceration	361	unknown	unknown	Y	N	N	N	2
Gastric Ulceration	32	causal	causal	Y	N	N	N	2
epigastric pain	31	possible	unknown	Y	N	N	N	Missing

*Day of onset is calculated from the treatment date.

^$^Assessed by the investigator.

^#^According to CTCAE grading.

AE, adverse event; ADE, device-related adverse event; REILD, radioembolization-induced liver disease; SADE, serious device-related adverse event; USADE, unanticipated serious device-related adverse event.

## 4 Discussion

This real-world, multi-center, retrospective study aimed at characterizing the safety and performance of ^166^Ho SIRT in routine clinical practice as opposed to prospective clinical trials.

Response and disease control rates both at and beyond three months vary in previous studies, and the results of the present study are within this range (13, 14, 16, 18, 19). Different magnitude of tumor response and disease control was achieved in different indications, with HCC patients having higher percentages of both than iCC and mCRC patients. That may suggest that SIRT yields different response dependent on the tumor type, possibly mediated through tumor vascularity and consecutive differences in absorbed dose. Median OS for mCRC and HCC are in line with results from HEPAR II, SIM and HEPAR Primary, respectively. Median OS found in the CIRT registry for mCRC and HCC treated with ^90^Y SIRT is similar, too. The RESIN registry showed a higher median OS for patients with mCRC treated with ^90^Y SIRT of 15.0 months (95% CI 13.3–16.9) compared to 8.9 months in the present study (95% CI 8.8–18). However, our results correspond to those of the pooled analysis of 2,517 patients from 23 studies reported by White et al. (20)in which weighted OS estimate was 9.6 months (95% CI 8.9–10.4). A relatively low number of patients receiving a liver transplantation after SIRT was recorded in this study. This may reflect the fact that the study included a more advanced patient population, possibly highlighting the context of SIRT usage in real-world practice. In patient cohorts with an earlier stage of disease, the proportion of patients qualifying for post-SIRT liver transplantation may be higher.

A dose-response-relationship of ^166^Ho SIRT has already been established (20). While the prediction of normal liver absorbed dose was accurate, the predicted tumor absorbed dose had been overestimated by approximately 30 Gy. This overestimation derived mainly from two centers, which contributed to a large number of the overall available datapoints. That could be possibly explained by dose calculation errors, such as partial volume effects, misregistration of CT and SPECT images. Saturation effects of the gamma camera, different catheter position between treatment planning and treatment, periprocedural occlusion of arterial branches and timepoint of dosimetry might contribute to the explanation of this finding. However, since accurate dose prediction is one of the advantages of ^166^Ho -SIRT in comparison to ^90^Y SIRT with ^99m^Tc-MAA for treatment planning, the overestimation of tumor absorbed dose should call attention to possible confounders.

Overall, the frequency of AEs reported in the present study is low compared to earlier ^166^Ho studies and is probably underreported (12, 13, 15, 21). Radioembolization induced liver disease (REILD) represents a rare but dangerous complication of SIRT and occurred in one patient (0.7%) with lethal outcome. Renal failure represents the only other lethal AE and was reported as possibly related to the procedure.

This study has several limitations. The retrospective nature and the single-arm design invariably contain limitations. The heterogeneous study population limits the comparison to previous studies. PFS (33.8%) and hPFS (37.9%) assessments had a high proportion of censored patients. Overall survival analysis had the highest proportion of censored patients (54.1%), which is in line with expectations: As survival analysis typically requires longer follow-up to observe the event of interest, the probability of a patient not to reach an event is high for patients treated at the end of the inclusion window. The retrospective design contains a risk of underreporting of AEs. However, AEs grade 4 or 5 according to CTCAE represent severe incidents, which are typically well documented in the patients’ health records. Therefore, it is reasonable to assume that the low frequency of severe AEs in this study can be interpreted as a sign of overall good tolerability of ^166^Ho SIRT. This is not only in line with previous ^166^Ho SIRT studies (12, 13, 15, 21), but also with recent preliminary results from the CIRT registry (22) and data from the RESIN registry (23), showing similar safety profiles for ^90^Y SIRT.

Another limitation of this study is the heterogeneity of imaging protocols for ^166^Ho microsphere treatments. Although the accuracy of dosimetry relies on imaging modality and reconstruction protocols (24, 25), standardization in imaging for ^166^Ho is lacking. We are convinced that imaging standardization will be paramount for achieving consistent and harmonized absorbed dose measurements. Meanwhile, the heterogeneity of imaging in our patient cohort reflects current real-world practice.

This observational study did not aim at comparing the treatment planning characteristics of ^99m^Tc-MAA and ^166^Ho Scout microspheres. However, published studies on this topic are available (9).

Finally, data granularity for specific questions is limited, for instance, the presence but not the degree of portal vein thrombosis was collected.

Strengths of this study include the high number of patients and the diversity of malignancies, treatment intentions and procedure characteristics. Data origin from multiple international centers further adds to the validity of the study.

Overall, this study confirms that the safety and effectiveness of ^166^Ho SIRT derived from prospective trials also applies in routine clinical practice. Treatment effectiveness of ^166^Ho SIRT was in line with treatment effectiveness reported in prospective Ho-166 SIRT trials as well as in large ^90^Y SIRT registries.

## Data availability statement

The raw data of this research are available from the corresponding author on reasonable request within the boundaries of the ethical committee approval. Requests to access the datasets should be directed to VS-Z, victor.schulze-zachau@usb.ch.

## Ethics statement

The studies involving humans were approved by the ethics committees of all participating centers. The studies were conducted in accordance with the local legislation and institutional requirements. The ethics committee/institutional review board waived the requirement of written informed consent for participation from the participants or the participants’ legal guardians/next of kin because this project consisted of retrospective collection of pseudonymized data in a patient cohort with a high mortality.

## Author contributions

VS-Z: Writing – review & editing, Writing – original draft, Visualization, Validation, Methodology, Investigation, Formal Analysis, Data curation. GV: Writing – review & editing, Investigation. PD: Writing – review & editing, Investigation. KD: Writing – review & editing, Investigation. FG: Writing – review & editing, Investigation. MH: Writing – review & editing, Investigation. RH: Writing – review & editing, Investigation. EB: Writing – review & editing, Investigation. RS: Writing – review & editing, Investigation. ML: Writing – review & editing, Investigation. JDM: Writing – review & editing, Investigation. RD: Writing – review & editing, Visualization, Resources, Project administration, Funding acquisition. CZ: Writing – review & editing, Validation, Supervision, Methodology, Investigation, Funding acquisition, Formal Analysis.
